# Ad Hoc Modeling of Rate-Dependent Adhesion in Indentation Relaxation Testing

**DOI:** 10.3390/ma17163944

**Published:** 2024-08-08

**Authors:** Ivan I. Argatov, Iakov A. Lyashenko, Valentin L. Popov

**Affiliations:** 1 Institut für Mechanik, Technische Universität Berlin, 10623 Berlin, Germany; ivan.argatov@campus.tu-berlin.de; 2 Center of Advanced Studies in Mechanics, Tribology, Bio- and Nanotechnologies, Samarkand State University, Samarkand 140104, Uzbekistan

**Keywords:** JKR-type adhesion, indentation testing, equilibrium interface energy, adhesion relaxation, ad hoc model, scaling method, minimal model

## Abstract

The phenomenon of rate-dependent adhesion has long been recognized as an intricate problem, and the so-far-developed physics and mechanics-based approaches resulted in analytical relations between the implicit form between the work of adhesion and the contact front velocity which are difficult to implement in practice. To address this issue in the framework of spherical indentation, the adhesion relaxation test in a nominal point contact is introduced to estimate the rate-dependent adhesion. Based on a stretched exponent approximation for the contact radius evolution with time, a relatively simple four-parameter model is proposed for the functional relation between the work of adhesion and the contact front velocity, and its fitting performance is compared to that of the known Greenwood–Johnson and Persson–Brener models.

## 1. Introduction

Adhesive properties of materials (in contrast to their bulk properties), being associated with surfaces, or to be more precise with interfaces, may strongly depend on the counterpart material [1,2,3]. In fact, the pull-off force of a sticky polymer measured with a metal or polymer spherical probe may differ by an order of magnitude [4,5]. Moreover, a prior repetitive loading/unloading of the adhesive contact or an extensive hold time in contact before the pull-off initiates is also known as one of the key factors affecting the adhesion strength [6,7,8]. The indicated issues may become decisive in soft robotics and soft-grip manipulators [9,10,11], where a rate-dependent loading protocol (e.g., ramp-like loading/unloading with an intermediate hold or periodic loading) is often used [12].

Sticky materials often exhibit pronounced viscoelastic properties [13,14] (in a wide range of operation frequencies of interest), and therefore, the adhesive contact model should account for the material bulk viscoelasticity. Still, both the viscoelastic bulk and the surface contributions to the adhesive component of the contact force depend on the indentation rate [15,16], which indirectly affects the contact front velocity, $\dot{a}$. Namely, in Refs. [17,18,19] there is a consensus that the velocity of the crack front (here, the opening outside the contact zone is treated as an annular crack propagating along a dissimilar contact interface) is a primary parameter that governs the variation in the work of adhesion, w.

Based on the analytical solution obtained by Schapery [20] for a mode I crack quasi-statically growing at the contact interface between different linear viscoelastic materials, a self-consistency model (in an implicit form involving an integral equation) was developed [21,22] for relating w to the shear creep compliance of the bulk material $\mu^{-1}\left(t\right)=\mu_{\infty }^{-1}\varphi \left(t\right)$, where $\mu_{\infty }$ is the equilibrium shear elastic modulus. In the case of a standard solid (with a single characteristic relaxation time, τ, and the ratio $\rho =\mu_{\infty }/\mu_{0}$ of the relaxation modulus $\mu_{\infty }$ to the instantaneous modulus $\mu_{0}$), Greenwood and Johnson [23] derived a simple analytical model that links w to the contact front velocity $\dot{a}=da/dt$, which in the opening mode (when $\dot{a}<0$) can be recast in the form

(1) $$\frac{\dot{a}}{\upsilon }=-\frac{w}{w_{\infty }}{\left[\ln\left(\frac{1-\rho }{1-w_{\infty }/w}\right)\right]}^{-1},$$

where *a* is the contact radius; υ is a certain characteristic velocity, which depends on τ among other model parameters; and $w_{\infty }$ is the equilibrium (thermodynamic) work of adhesion (measured in a quasi-static process such that $|\dot{a}|/\upsilon \ll 1$).

Other analytical approaches for establishing relations between w to $\dot{a}$ were developed by de Gennes [24] and Persson and Brener [25], though no simple analytical relations similar to (1) have been found for a standard viscoelastic solid model so far. In particular, for a standard solid, the Persson–Brener model takes the form of the following implicit equation [26]:

(2) $$\frac{w}{w_{\infty }}={\left\{1-\left(1-\rho \right)\left(\sqrt{1+{\left(\frac{w_{\infty }}{w}\frac{|\dot{a}|}{\upsilon }\right)}^{-2}}-{\left(\frac{w_{\infty }}{w}\frac{|\dot{a}|}{\upsilon }\right)}^{-1}\right)\right\}}^{-1}.$$

On the other hand, the implicit relation (1) is not at all popular for fitting experimental data, and the overwhelming majority of studies with modeling experiments on rate-dependent adhesion [27,28,29,30] employ the following phenomenological model introduced by Gent and Schultz [31]:

(3) $$w=w_{\infty }\left(1+{\left(\frac{|\dot{a}|}{\upsilon }\right)}^{\beta }\right).$$

It is noted here that Formulas (1) and (3) each contain only three fitting parameters.

While this direct approach is technically sound (fitting Formula (3) to the experimental data is much simpler than Formula (1)), the use of Formula (3) has certain limitations.

Moreover, as was argued by Shull [32], since in many cases it is impossible to separate the bulk and surface contributions to the adhesion strength, it is recommended to make use of Equation (3) with parameters determined from experiment. In particular, the Gent–Schultz law (3) predicts an unlimited growth in w with increasing crack velocity, which contradicts the Greenwood–Johnson model (1), predicting that w is limited by the value $w_{\infty }/\rho$ (as the argument of the logarithm in Equation (1) approaches the unit value in the regime of high-rate detachment). However, the latter model is rather stiff to accurately fit diverse experimental data.

As both the Greenwood–Johnson (GJ) model and the Persson–Brener (PB) model are based on the standard linear solid model, which is applicable for a very narrow band of frequencies in characterizing the strain relaxation kinetics, generally speaking, one cannot expect the GJ and PB models to reliably reproduce adhesion experimental data, unless by chance. However, a significant difference between fitting approaches in viscoelasticity and rate-dependent adhesion is that the viscoelastic standard solid model can be easily generalized, for example, by employing mechanical spring–dashpot models or the traditional Prony series approach. In contrast to the viscoelastic case, while both the GJ and PB models stem from the general model developed by Schapery [20], to the best of the authors’ knowledge no other models for describing the rate dependency of adhesion have been published in the literature that are analogous to viscoelastic models more general than the standard solid model.

Thus, there is an urgent need for developing a flexible phenomenological model (relating w to $\dot{a}$) as well as for a designing a benchmark indentation test, which allows for evaluation of the rate-dependent adhesive properties of engineering adhesives.

## 2. Main Result

An evident weakness of the analytical relations (1) and (2) is that they are stiff, in the sense that, while both the general Greenwood–Johnson [23] and Persson–Brener [25] models are potentially flexible, their specific realizations lack adjustability to handle diverse rate-dependent adhesion data. This is because both Formulas (1) and (2) originate from a three-parameter standard solid model. In the case of adhesion, the meaning of the parameter ρ is determined by the limit $w\to w_{\infty }/\rho$ as $|\dot{a}|\to \infty$. In other words, in contrast to the Gent–Schultz law (3), both Formulas (1) and (2) predict that the ratio $w/w_{\infty }$ belongs to the interval $\left(1,\rho^{-1}\right)$ for any non-zero contact front velocity.

To overcome the flexibility issue, one strategy is to introduce at least one additional (dimensionless) fitting parameter. In the present study, our ad hoc analysis of the adhesion relaxation test yields the result

(4) $$\frac{\dot{a}}{\upsilon }=-\alpha \left({\left(\frac{w}{w_{\infty }}\right)}^{1/3}-1\right)\ln^{(\alpha -1)/\alpha }\left(\frac{\rho^{-1/3}-1}{{\left(w/w_{\infty }\right)}^{1/3}-1}\right),$$

where we have used the notation $\ln^{\sigma }\left(x\right)={\left(\ln x\right)}^{\sigma }$.

We note that Formula (4) contains four parameters: $w_{\infty }$, υ, ρ, and α. Namely, an additional (fourth) parameter α equips the formula with a greater flexibility than the three-parameter Formula (1).

**Figure 1:**
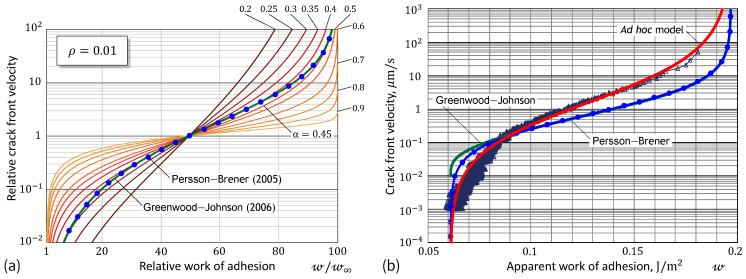
(**a**) Flexibility of the ad hoc adhesion constitutive model in comparison with the Greenwood–Johnson model (1) and the Persson–Brener model (2). (**b**) Fitting the ad hoc model to the adhesion relaxation data [23,25].

Figure 1a demonstrates the high flexibility of our Formula (4) compared to that of the specific Greenwood–Johnson [23] (1) and Persson and Brener [25] (2) models. It is of interest to note that Formula (4) contains only two fitting constants (namely, ρ and α), if it uses the relative parameters $\dot{a}/\upsilon$ and $w/w_{\infty }$ as references. The maximum value of the ratio $w/w_{\infty }$ coincides with the value of 1/ρ. That is why Figure 1a exhibits the full parametric analysis of our model. Figure 1b shows the fitting performance of the so-called ad hoc model (4) for a set of data obtained from the spherical indentation.

## 3. Methods

### 3.1. JKR-Type Rate-Dependent Adhesion

We consider a typical indentation test performed with a rigid indenter of axisymmetric profile, keeping in mind the case of a spherical indenter of radius *R*. Under the action of an external normal (i.e., vertical) force, *P*, the indenter receives some displacement, δ, which is measured from the unperturbed surface of a tested elastic sample. In the special case of a flat-ended cylindrical indenter of radius *a*, the circular contact area does not change during the indentation and the contact force/displacement relation is linear, that is, P=S(a)δ, where S(a) is the indentation stiffness [33].

For a convex indenter (e.g., spherical or conical), the effect of adhesion modifies the force/displacement relation because the adhesion increases the contact area compared to non-adhesive contact. In the latter case, the contact force and the indenter displacement, as functions of the contact radius, are denoted by $P_{\circ }\left(a\right)$ and $\delta_{\circ }\left(a\right)$, respectively.

By using energy considerations, the Johnson–Kendall–Roberts (JKR) model of frictionless contact is generalized as follows [34,35,36]:

(5) $$\frac{S^{\prime }\left(a\right)}{4\pi a}\frac{{\left(P_{\circ }\left(a\right)-P\right)}^{2}}{S^{2}\left(a\right)}=w,\;\frac{S^{\prime }\left(a\right)}{4\pi a}{\left(\delta_{\circ }\left(a\right)-\delta \right)}^{2}=w,$$

Here, $S^{\prime }\left(a\right)$ is the derivative of S(a), that is, $S^{\prime }\left(a\right)=dS\left(a\right)/da$, and w is the work of adhesion.

Following Maugis and Barquins [37], Equation (5) can be extended for the case of rate-dependent adhesion by regarding w as the apparent work of adhesion such that $\left(w-w_{\infty }\right)/w_{\infty }=\varphi \left(\dot{a}\right)$, where $w_{\infty }$ is the thermodynamic (equilibrium) work of adhesion, and φ is a dimensionless function of contact front velocity $\dot{a}$, defined as the derivative of the contact radius *a* with respect to the time variable *t*. The function φ is supposed to be independent of the contact geometry of the system (specifically, independent of the indenter geometry), while the temperature effects can be accounted for via the temperature shift factor. For decreasing ($\dot{a}<0$) and increasing ($\dot{a}>0$) contacts, the ratio $\left(w-w_{\infty }\right)/w_{\infty }$ takes positive values below and above 1, respectively, and, of course, φ(0)=0. In particular, in the case of decohesion ($\dot{a}<0$), the phenomenological Gent–Schultz law [31] has the form $\varphi \left(\dot{a}\right)=\left(\right|\dot{a}{\left|/v\right)}^{\beta }$, where *v* is a characteristic velocity and β is a dimensionless constant.

To be more specific we need analytical expressions for the functions S(a), $P_{\circ }\left(a\right)$, and $\delta_{\circ }\left(a\right)$ that enter Equation (5). Let the tested sample be modeled as an elastic isotropic layer of thickness *h*. Then, we can write that

(6) $$S\left(a\right)=2aE^{*}\kappa \left(\frac{a}{h}\right),$$

where $E^{*}=E/\left(1-\nu^{2}\right)$ is the reduced elastic modulus (with Young’s modulus, *E*, and Poisson’s ratio, ν), κ(ε) is the so-called indentation scaling factor, and ε=a/h is the relative contact radius.

In the case of frictionless contact, the scaling factor κ(ε) satisfies the normalization condition κ(0)=1 and depends on the layer’s Poisson’s ratio ν as well as on the boundary conditions imposed on the layer’s bottom surface. Concomitantly, the Hertzian solution can be generalized as

(7) $$P_{\circ }\left(a\right)=\frac{4a^{3}}{3R}E^{*}f_{P}\left(\frac{a}{h}\right),\;\delta_{\circ }\left(a\right)=\frac{a^{2}}{R}f_{\delta }\left(\frac{a}{h}\right),$$

where $f_{P}\left(\varepsilon \right)$ and $f_{\delta }\left(\varepsilon \right)$ are the force and displacement scaling factors, respectively, for which we have asymptotic [38] and analytical [39,40] approximations.

In light of Equations (6) and (7) right, we can rearrange Equation (5) left as

(8) $$\frac{4a^{3/2}}{3R}f_{P}\left(\varepsilon \right)k\left(\varepsilon \right)=\frac{1}{E^{*}}\frac{k(\varepsilon )P}{a^{3/2}}+\sqrt{\frac{8\pi w}{E^{*}}},$$

where $k\left(\varepsilon \right)=\sqrt{\kappa \left(\varepsilon \right)+\varepsilon \kappa^{\prime }\left(\varepsilon \right)}/\kappa \left(\varepsilon \right)$, and $\kappa^{\prime }\left(\varepsilon \right)$ is the derivative of the scaling factor κ(ε), that is, $\kappa^{\prime }\left(\varepsilon \right)=d\kappa \left(\varepsilon \right)/d\varepsilon$.

Equation (8) generalizes the scaling relation of the JKR model used previously [41,42,43] for the experimental evaluation of the equilibrium work of adhesion $w_{\infty }$.

**Figure 2:**
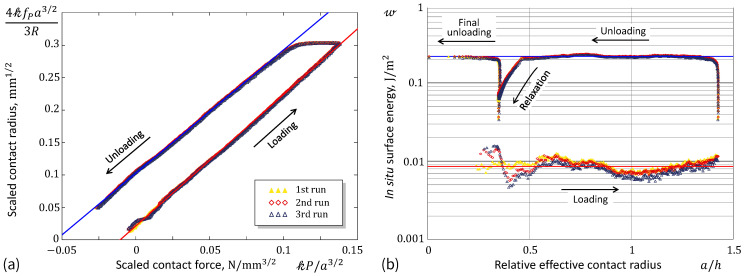
(**a**) Experimental data and fitting lines according to the generalized JKR model. (**b**) In situ work of adhesion and the fitting results.

By the method of linear regression, Equation (8) can be applied for estimating the effective work of adhesion w under the condition of constant velocity indentation (advancing and receding), which approximately ensures a constant contact front propagation. Figure 2a illustrates the application of the method using a set of experimental data obtained in a constant-rate spherical indentation (see the next section). The analytical solution for a bonded incompressible layer [39] was used for evaluation of the scaling factors that enter the regression Formula (8). It should be noted that the adhesion fluctuations observed in Figure 2b (see the loading branch) can apparently be explained by adhesion heterogeneity [44,45,46,47], the indenter’s surface roughness [48,49,50], or imperfections on the tested sample’s surface [51,52]. However, what is more important to note is that the surface energy drops by almost an order of magnitude during the relaxation, when the indenter does not move.

When the elastic modulus *E* is known (for ν=0.5 we have $E^{*}=\left(4/3\right)E$), Equation (5) can be used for a posteriori estimation of the in situ surface energy (see Figure 2b).

It should be made clear that the adhesion relaxation test at the position of nominal contact is well fitted by the linearly elastic JKR model, and the viscoelasticity of the bulk material can be ignored as negligible. In principle, Equation (5) can be generalized for viscoelastic materials (see, e.g., [53,54]), but it remains an open question how to account for the thickness effect [55] in spherical indentation of adhesive viscoelastic materials with the rate-dependent adhesion.

### 3.2. Spherical Ramp Indentation

**Figure 3:**
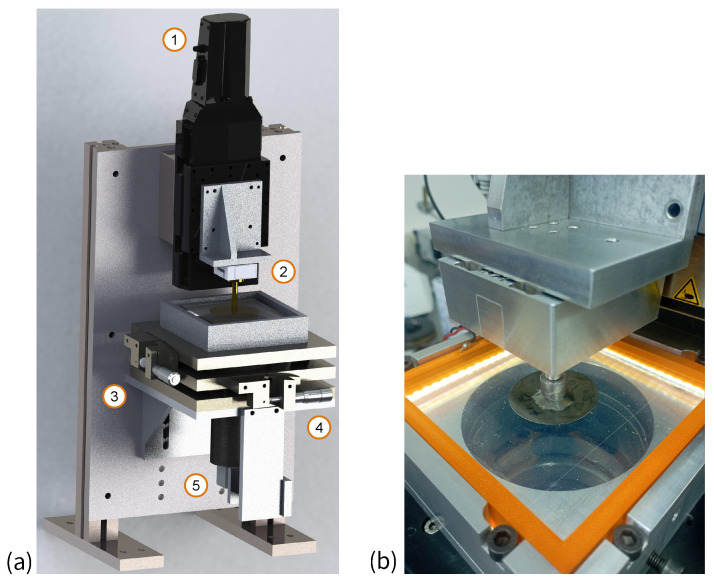
(**a**) Schematic of the experimental equipment: (1) PI L-511.24AD00 driver (Physik Instrumente (PI) GmbH & Co. KG, Karlsruhe, Germany), (2) three-axis force sensor ME K3D40 (ME-Meßsysteme GmbH, Hennigsdorf, Germany), (3) and (4) tilt mechanisms, (5) USB digital camera; (**b**) close-up view of the spherical indenter contact with a transparent adhesive rubber-like gel elastomer (thermoplastic polystyrene-type gel TANAC CRG N3005).

In the indentation experiments (see Figure 3a), a spherical steel indenter with radius R=100mm was indented into a layer of transparent adhesive rubber of thickness h=5mm to a predefined depth $\delta_{\max}=0.3\;\mathrm{mm}$, after which it was immediately (that is without any delay) lifted to the zero level δ=0. At this level, the indenter remained at rest for a duration of two hours, after which it was pulled off from the rubber layer until the moment of loss of contact. The movement speed of the indenter in both directions (loading and unloading) was 1μm/s. The optically transparent rubber used allows the determination of the in situ contact area (see Figure 3b). Throughout all the experiments (three trials), the values of the normal contact force *P*, as well as photographs of the contact area, were recorded at one-second intervals. The mechanical (elastic) and physical (adhesive) properties of the tested material (thermoplastic polystyrene-type gel TANAC CRG N3005, produced by TANAC Co., Ltd., Gifu, Japan) were measured during the experiment. The linear elasticity framework was used to describe the material’s behavior under indentation. Mathcad software version 14 was employed for the numerical computations involved in analyzing the experimental data. A detailed description of the experimental setup is given elsewhere [56].

**Figure 4:**
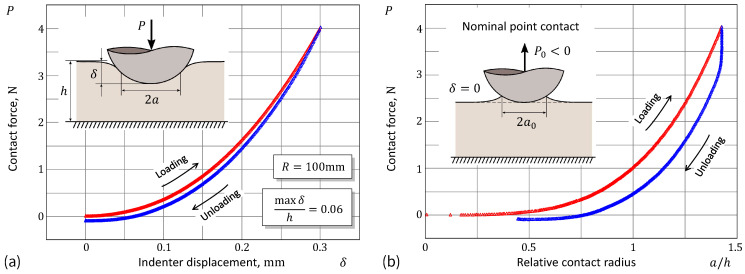
Experimental data from spherical indentation: (**a**) Force–displacement curve and (**b**) force/contact radius relation under a constant velocity, displacement driven ramp-like loading/unloading test.

Figure 4a shows a typical force–indentation curve, which consists of two branches (loading and unloading). The corresponding relation between the contact force and the contact radius is shown in Figure 4b. The use of the relative contact radius ε=a/h reveals the fact that the classical JKR theory cannot be applied to the stiffness analysis over the entire range of indentation depths due to the significant influence of the thickness effect near the maximum indentation.

**Figure 5:**
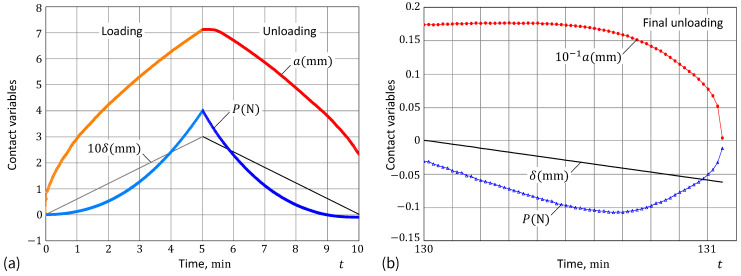
Variation in the contact variables with time (**a**) in the initial two-phase (loading/unloading) stage and (**b**) in the final unloading stage.

Figure 5a presents the time-dependent evolution of the contact variables during the two-stage ramp loading. The final unloading stage (after the two-hour hold period) is shown in Figure 5b.

In this study, spherical indentation into a layer of adhesive rubber was conducted. Therefore, we need to address the JKR-type rate-dependent adhesion for a specimen of finite thickness, as the classical JKR model is typically applied to half-space materials. However, by modeling the tested sample as an elastic half-space, we can discard the force and displacement scaling factors in Equation (7) and directly substitute the classical Hertzian solution into Equation (5) to yield Equation (9). This raises the question: Why not conduct the indentation test on a half-space material?

The answer is simple: large contact areas are required to improve the accuracy of determining the contact radius from photo images of the contact spot. Therefore, technical issues, such as video camera resolution and the thickness of commercially available adhesive material, prompted the extension of the classical JKR theory. Additionally, in many engineering applications, the adhesive effect must be accounted for in indentation of thin coatings. Thus, the developed theoretical framework enables the analysis of quasi-static tests under conditions similar to those encountered in real-world engineering applications.

It should also be emphasized that in indentation testing, we measure the contact force as an *integral* reaction of the adhesive material by summing up the contact reactions over the entire contact area as well as the attractive forces acting at the periphery of the contact; that is, by including the bulk contribution due to the viscoelastic resistance to deformations and the surface contribution due to adhesion. That is why we minimize the bulk contribution by restricting our attention to the adhesion relaxation test in the position of nominal point contact, where the contact is maintained solely by the adhesive forces.

### 3.3. Adhesion Relaxation

We consider a displacement-controlled loading protocol such that at a time moment $t=t_{0}$ the indenter movement stops, that is, $\delta_{0}\left(t\right)=\delta_{0}\left(t_{0}\right)$ for $t\geq t_{0}$. Then, the contact enters a state, called the adhesion relaxation, where the contact force P(t) and the contact radius a(t) continue to vary due to the time-dependent properties.

**Figure 6:**
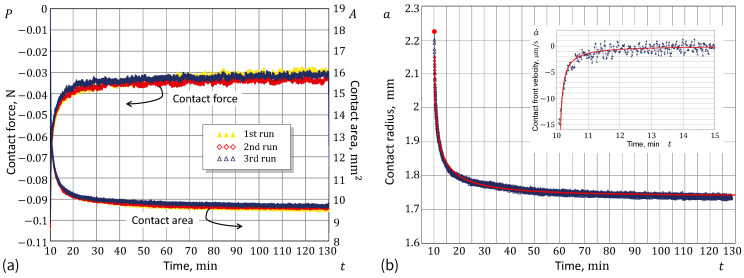
(**a**) Experimental data from the adhesion relaxation test: Contact force (left ordinate) and contact area (right ordinate) as functions of time. (**b**) Fitting the adhesion relaxation experimental data: Contact radius (main plot) and contact front velocity (insert plot) as functions of time.

In Figure 6a, the dependencies of the contact area *A* and the normal contact force *P* on time *t* are shown for the resting stage at a nominal point contact (for the indentation depth δ=0), where the contact exists solely due to adhesion (thus, the normal force *P* is negative here). Three consecutive indentation cycles were conducted in the experiment, which are depicted in the panels of Figure 6 with different colors. The time-dependent reduction in the contact area (or equivalently in the contact radius $a=\sqrt{A/\pi }$) leads to a gradual decrease in the adhesive component of the contact force, and, as a result, a time-dependent increase in the measured normal force *P*.

The adhesion relaxation in a nominal point contact configuration, when $\delta_{0}\left(t_{0}\right)=0$, attracts a special interest for reasons of simplicity of interpretation. To further simplify the consideration we can neglect the sample thickness effect, so that Equation (5), in view of (7), reduces to the classical JKR model.

So, provided that $\delta_{0}\left(t_{0}\right)=0$ for $t\geq t_{0}$, Equations (5) right and (7) right, under the assumption that a≪h, yield the relation

(9) $$\frac{E^{*}}{2\pi }\frac{a^{3}}{R^{2}}=w,\;t\geq t_{0}.$$

It should be underlined here that Equation (9) follows from the JKR Equation (5) right and the Hertzian Equation (7) right, both of which are derived in the framework of linear elasticity. The nonlinear case (large deformations) not only requires special consideration but also complicates the solution of the inverse problem of adhesion law identification.

The contact radius *a*, monitored as a function of time, decreases from some initial value $a_{0}=a\left(t_{0}\right)$ to the equilibrium value $a_{\infty }=\lim a\left(t\right)$ as t→∞. Correspondingly, Formula (9) directly provides the respective variation in the effective work of adhesion w as a function of time. However, our primary interest lies in establishing a functional relation between w and the contact front velocity $\dot{a}\left(t\right)$. The latter can be achieved graphically, by plotting the calculated value of w(t) according to Equation (9) against the numerically evaluated value of $\dot{a}\left(t\right)$ from the experimentally collected data for a(t).

The major reason for the difficulty in the practical implementation of the above algorithm is the fact that the optically collected data for the contact area A(t) are very noisy, and therefore, generally we do not attempt to directly evaluate the derivative $\dot{a}\left(t\right)$ from $\sqrt{A(t)/\pi }$ (see the insert in Figure 6b). In order to cope with the noise issue, the use of a smooth approximation for $a\left(t\right)=\sqrt{A(t)/\pi }$ has been proposed to obtain the effective velocity $\dot{a}\left(t\right)$ from the derivative of the analytical fitting formula for a(t).

### 3.4. Ad Hoc Fitting Model

We start by noting the fact that the initial contact front velocity is almost infinite (see Figure 6b). This is approximately observed experimentally and has been supported theoretically [23] in the case of contact under zero load. After a few trials, the following simple fitting approximation was acquired:

(10) $$a=\left(a_{0}-a_{\infty }\right)\exp\left\{-{\left(\frac{t-t_{0}}{\tau }\right)}^{\alpha }\right\}+a_{\infty }.$$

This is a so-called [57] stretched-exponential law. It is important to note here [58] that caution should be exercised when assigning a physical meaning to the stretched exponent α.

Formula (10) contains four fitting parameters $a_{0}$, $a_{\infty }$, τ, and α. The initial condition $a\left(t_{0}\right)=a_{0}$ implies that α>0. Differentiation of both sides of Equation (10) with respect to *t* yields

(11) $$\dot{a}\left(t\right)=-\frac{\alpha (a_{0}-a_{\infty })}{\tau }{\left(\frac{t-t_{0}}{\tau }\right)}^{\alpha -1}\exp\left\{-{\left(\frac{t-t_{0}}{\tau }\right)}^{\alpha }\right\}.$$

The limit condition $\dot{a}\left(t\right)\to -\infty$ as $t\to t_{0}+0$ implies that α<1, and therefore, we have α∈(0,1).

Now, we observe that from Equation (10) it follows that $\left(t-t_{0}\right)/\tau =\ln^{1/\alpha }\left[\left(a_{0}-a_{\infty }\right)/\left(a-a_{\infty }\right)\right]$, so that Formula (11) can be represented in the form

(12) $$\dot{a}\left(t\right)=-\frac{\alpha (a\left(t\right)-a_{\infty })}{\tau }\ln^{(\alpha -1)/\alpha }\left(\frac{a_{0}-a_{\infty }}{a\left(t\right)-a_{\infty }}\right).$$

Thus, Equations (9) and (12) provide the adhesion constitutive relationship in an implicit parametric form. To represent it in the final form, we note that according to Equation (9) we have $a_{\infty }^{3}=\left(2\pi R^{2}/E^{*}\right)w_{\infty }$, and thus, Equation (9) can be recast as follows: ${\left(a/a_{\infty }\right)}^{3}=w/w_{\infty }$. Finally, by introducing the auxiliary fitting parameters $\upsilon =a_{\infty }/\tau$ and $\rho ={\left(a_{\infty }/a_{0}\right)}^{3}$, we can rewrite Equation (12) in the nondimensionalized implicit form (4). It is remarkable that Equation (4) can be interpreted as a *minimal* model [59], as it captures the essential phenomenology of rate-dependent adhesion.

## 4. Discussion and Conclusions

We start with pointing out that our fitting model is of the same complexity as the Greenwood–Johnson model (1), while being more flexible due to an additional degree of freedom (see Figure 1a). In principle, Formula (1) can be modified by introducing an additional fitting parameter, e.g., by analogy with our Formula (4). While this undoubtedly increases its flexibility, such a generalization loses a direct connection to the viscoelastic standard solid model, which depreciates this phenomenological approach. On the other hand, when applying a stretched-exponential law for the creep compliance function $\varphi \left(t\right)=1-\left(1-\rho \right)\exp\{-{\left(t/\tau \right)}^{\alpha }\}$ in the general Greenwood–Johnson model, we arrive at the following result:

(13) $$\frac{\dot{a}}{\upsilon }=-\frac{w}{w_{\infty }}{\left[\ln\left(\frac{1-\rho }{1-w_{\infty }/w}\right)\right]}^{-1/\alpha }.$$

We leave the comparison of the fitting capabilities of Formulas (13) and (4) outside the scope of the present study. However, what is more interesting is to learn from experimental testing whether the used rubber-like gel material possesses rheological properties [60,61] that are well described by the stretched exponent.

As can be seen with the naked eye in Figure 1b, the proposed ad hoc formula works better than the GJ and PB models, because it includes an additional fitting parameter, which allows the slope of the fitting curve to change, as is evident in Figure 1a. This model’s flexibility can be conveniently exploited for improving the fit of experimental data. Moreover, the ad hoc approach enhances the scientific understanding of the problem, as it indirectly suggests that simple exponential surface kinetic modeling (see, e.g., [62,63]) is insufficient to account for non-exponential adhesive behavior.

It is interesting that by adopting a Prony’s series approximation for the shear relaxation modulus, the introduction of an additional exponential term to the standard solid model brings two additional fitting constants. Thus, the corresponding refined Greenwood–Johnson model contains five independent parameters. Yet, a warning about the use of exponential approximations with cross-correlated parameters [64] is important for curve fitting stability.

The PB model can, in principle, be generalized to a power-law viscoelastic material model. Compared to the standard solid model, this would add one more fitting parameter, providing more freedom to better fit the experimental data. However, such an approach would hardly be worth the effort, as the PB modeling leads to the law of adhesion in an implicit form (indeed, the same parameter w appears on both sides of Equation (2)).

Experimentalists encounter challenges with the GJ and especially PB models because the contact front speed is not a controllable parameter in indentation experiments. Given a specific indentation or retraction rate, the corresponding effective surface energy, which is rate-dependent and generally unknown a priori, needs to be determined (and the contact-front speed must also be measured). In contrast, the ad hoc modeling approach focuses on accurately fitting the evolution of the contact radius. This approach aims not only to describe the contact front speed analytically but also to capture its dynamic behavior more effectively.

A note should be made about the comparison of the Greenwood–Johnson (1) and Persson–Brener (2) models, which both originate from the viscoelastic standard solid model. It can be easily verified that from Equations (1) and (2), respectively, it follows that

(14) $$\frac{1-\rho }{1-w_{\infty }/w}=\exp\left(\frac{w}{w_{\infty }}\frac{\upsilon }{|\dot{a}|}\right),\;\frac{1-\rho }{1-w_{\infty }/w}=\sqrt{1+{\left(\frac{w}{w_{\infty }}\frac{\upsilon }{|\dot{a}|}\right)}^{2}}+\frac{w}{w_{\infty }}\frac{\upsilon }{|\dot{a}|}.$$

Thus, the difference between the predictions of the two models (14), which can be observed in Figure 1b, is a direct consequence of the approximation $\exp\left(x\right)\approx \sqrt{1+x^{2}}+x$ that works well only for 0≤x<1. However, as is seen in Figure 1a, the outputs of both models practically completely coincide outside the interval of almost quasi-static loading. Yet, it goes without saying that the implementation of Equation (1) is much simpler than that of Equation (2).

**Figure 7:**
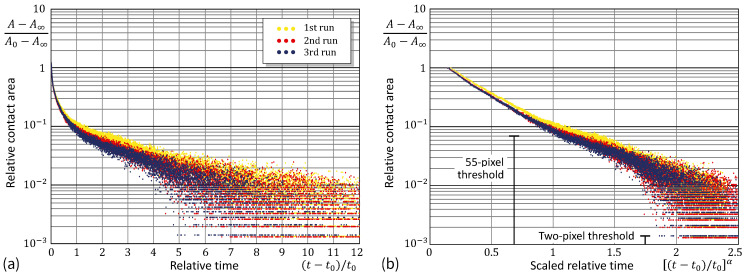
Experimental data from the relative contact area in adhesion relaxation: test in the logarithmic ordinate scaling (**a**) and with the additional power abscissa scaling (**b**).

**Figure 8:**
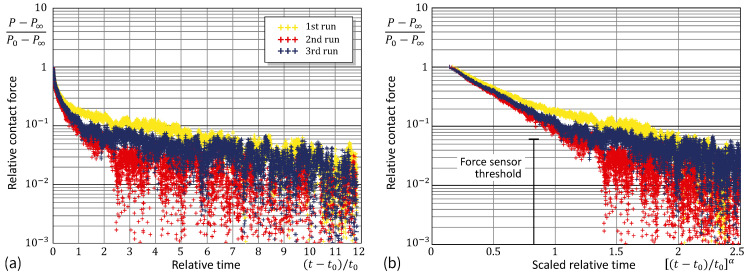
Experimental data from the relative contact force in the adhesion relaxation: test in the logarithmic ordinate scaling (**a**) and with the additional power abscissa scaling (**b**).

Observe that in many cases decay evolution may exhibit an exponential character, so that by plotting the time dependence using a logarithmic ordinate, the graph can be transformed into a straight line. Figure 7a and Figure 8a show that the exponential law is not adequate in the case under consideration. On the other hand, by additionally stretching the time coordinate (in the same way as that recovered in the analysis of the contact radius variable), we see from Figure 7b and Figure 8b that the stretched-exponential law works not only for the contact area (as could be expected) but also for the contact force. It is interesting to note here that, in the main, the equation $P=\left(P_{0}-P_{\infty }\right)\exp\left\{-{\left[\left(t-t_{0}\right)/\tau \right]}^{\alpha }\right\}+P_{\infty }$ holds up until the stage of ‘dynamic equilibrium’, where the contact force fluctuations are within the range of the force sensor threshold.

Further, it should be noted that the contact area was calculated from the snapshot images by counting the pixels classified as belonging to the contact by checking the pixel brightness. (The discrete nature of the contact area variation is readily seen in Figure 7). In our experimental data, the linear size, Δx, of the contact patch corresponding to one pixel was 85/1337mm. For the contact radius a=1.73mm (see Figure 6b), the area difference $\pi {\left(a+\Delta x\right)}^{2}-\pi a^{2}$ approximately equals the total area of 55 pixels. Thus, when the contact area variable falls below this threshold (see Figure 7b), the contact nears dynamic equilibrium.

In principle, Formula (4) can be utilized for formulating the adhesion law in the loading stage (when the contact radius increases). However, a much more justified approach would be to consider an adhesive creep test under zero load. In such a case, the JKR model yields the following result in the implicit form

(15) $$\frac{\dot{a}}{\upsilon }=F\left(\frac{w}{w_{\infty }}\right)$$

of adhesion law (1) and (4), which is convenient in numerical solving the indentation problems. Indeed, depending on the type of loading control (that is, force-controlled or displacement-controlled indentation), the generalized JKR Equation (5) should be used.

For instance, in the displacement controlled test (where the indenter displacement δ(t) is a given function of time *t*), Equation (5), in view of (7) right, leads to the differential equation

(16) $$\frac{da}{dt}=\upsilon F\left(\frac{S^{\prime }\left(a\right)}{4\pi aw_{\infty }}{\left\{\frac{a^{2}}{R}f_{\delta }\left(\frac{a}{h}\right)-\delta \left(t\right)\right\}}^{2}\right),$$

which is solved explicitly with respect to the time derivative of the contact radius.

In our analysis, we adopted the stretched-exponential variation (10) for the contact radius evolution as the best fit for the acquired experimental data as a result of going through different options including the approximation $\left(a-a_{\infty }\right)/\left(a_{0}-a_{\infty }\right)={\left\{1+{\left[\left(t-t_{0}\right)/\tau \right]}^{\alpha }\right\}}^{-1}$. Clearly, each approximation may lead to a more accurate fitting formula for the adhesion law for other types of time-dependent adhesive materials.

It should be noted that the $\dot{a}$ vs. w data in Figure 1b were recovered from the adhesion relaxation test in a fixed nominal point contact, where the contact front velocity varies in an interval over a few orders of magnitude. Still, this interval is rather short for expanding the range of validity of Formula (4) to high-rate pull-off tests.

To conclude, the ad hoc introduced fitting model (work of adhesion as a function of contact front velocity) for the JKR-type rate-dependent adhesion has been shown to be quite effective in capturing the variation of the adhesive properties of adhesive rubber-like gel materials.

## Data Availability

The original contributions presented in the study are included in the article, further inquiries can be directed to the corresponding authors.
